# Supplementary material for: Reliability of a convolutional neural network in segmenting multiple sclerosis lesions from MRI: Impact of data augmentation, image modality and tolerance with U-Net architecture
Source: PLOS Digit Health. 2026 Apr 1;5(4):e0001316. doi: 10.1371/journal.pdig.0001316 (PMC13042652; doi:10.1371/journal.pdig.0001316)
Supplement: S1 Text — (PDF) [file pdig.0001316.s001.pdf]

Supplementary Materials

S1 Text: U-Net Architecture

The input layer of U-Net is followed by two convolution layers. In the convolution process (for each layer) a kernel/filter of dimensions 3×3 passes over the input image using a stride of one, reducing the dimensions of the image by two voxels. This can be seen in Fig 3 in which the dimensions of the example input image produce feature maps which incrementally reduce in size ((input) 128×128 to (f-map1) 128×128 to (f-map2) 128×128). Padding, however is used to keep the dimensions consistent in any convolutional block. The filter/kernel contains randomly initialised weights. Scalar dot products are performed between all values in the kernel and the section of the image it passes over during the process. The values are summed and then outputted as a single value onto the feature map. In the process, the nine randomly initialised weights comprising the filter are trained to recognise features of interest from the set of training images. These weights are trained using backpropagation which depends on a loss function to calculate an error gradient which is then used to proliferate adjustments in the weights across the entire neural network. The activation function used is known as a rectified linear unit (ReLU), which replaces all negative values with 0 and leaves all positive values unchanged (in the feature map).

In the second convolution layer, a kernel acts on the set of feature maps (tensor) which are simultaneously produced. This is achieved by the kernel containing an extra depth dimension which is equal to the number of inputted feature maps. As previously, dot product operations between the kernel passing over the image and the image are completed but this time produce a stack of outputs which are then aggregated to reduce the output depth dimension to one. Each kernel used performs this operation followed by an aggregation, this prevents the number of feature maps exploding in number whilst also retaining information of importance within the image. It should also be noted that if the values in any position on the feature map exceeds 255 they are truncated (capped).

After the two initial convolution layers, a max-pooling layer is used. The size of the kernel for this phase is 2×2 kernel/filter, and a step of two is implemented. This simply takes the maximum pixel value of every four values and replaces all values with a single one, this being the highest in the kernel thereby reducing size of the image by a factor of four whilst retaining features of interest.

A dropout layer follows to prevent overfitting thus improving generalisation by reducing the impact of dominant features. Setting the value at 0.5 in this layer leads to 50 percent of the values in the outputted feature map becoming 0 on a stochastic basis. The contracting path described, as a single entity, is essentially an encoder, condensing the information into a smaller matrix/tensor than before and eliminating perceived redundant information whilst retaining and amplifying features of significance. In the original architecture, this process repeats three times, iteratively contracting the image whilst preserving features of interest. Finally, a two layer convolution with a pooling layer absent is implemented.

During the expansion or the upsampling phase of the processing of the neural network, feature maps from the contracting path are concatenated to help with feature extraction. This assists with preserving the memory of features extracted earlier in the neural network, and ultimately makes the network more generalisable and helps to prevent overfitting. Padding is introduced to ensure that the dimensions of feature maps at any one point in the neural network are uniform. Padding simply enlarges a feature map by appending zeros to the perimeter of a matrix. The final stage in the process, prior to generating the output, is the use of a sigmoid function (see equation 1) as the activation function, which scales the output

values within the matrix to ensure that they all fall between 0 and 1.

$$Output = \frac{1}{1 + e^{-x}} \tag{1}$$

An input of 0 causes the output to yield 0.5. and a very large positive number yields 1 and a very large negative number yields 0. This is followed by the decoding or up-sampling process and involves deconvolution, which re-expands the feature maps. In the deconvolution process, a filter is passed over the set of images and the dot product is performed between a 2×2 kernel and each pixel/voxel, leading to four pixels being produced for every pixel, doubling the dimensions in both width and length and the area of the image by four. The final element used within the U-Net architecture are skip connections, which concatenate feature maps from earlier stages in the process. This allows any information lost in the encoding process to be re-inserted into the training process permitting the neural network to extract a wider variety of features.
