# Supplementary material for: Reliability of a convolutional neural network in segmenting multiple sclerosis lesions from MRI: Impact of data augmentation, image modality and tolerance with U-Net architecture
Source: PLOS Digit Health. 2026 Apr 1;5(4):e0001316. doi: 10.1371/journal.pdig.0001316 (PMC13042652; doi:10.1371/journal.pdig.0001316)
Supplement: S2 Text — Table A in S2 Text includes further results with the data redistributed between the training in the testing sets, breaking the rubric of the published challenge. Instead 38 or 43 brain volumes for training and 15 or 10 brain volumes for testing were used. Each imaging modality was looked at and each test was conducted 10 times. Table B in S2 Text contains data on the impact of removing the impact of a defective mask (patient 26) which contained no imaging mask in the set of ground truth data used. Table C in S2 Text contains results utilising different imaging modalities for both testing and training, examining every permutation (25 versions) each tested 10 times with an average taken. (PDF) [file pdig.0001316.s002.pdf]

S2 Text: Supplementary Analysis

Within the S2 text is contained supplementary data outside of the scope of the initially planned four phases of experimentation.

Table A in S2 Text. *A comparison of mean Dice results after inverting the training and testing sets and redistributing the data such that the training–testing volume split was 43:10.*

Supplementary data 1										
Modality	Ratio	Dice	Control	p-value	$p \lesssim 0.05$	Min	q1	Median	q3	Max
T1	38:15	0.5970	0.5747	0.0007	✓	0.5902	0.5966	0.5981	0.5999	0.6001
T2	38:15	0.5982	0.5744	0.0027	✓	0.5916	0.5950	0.5967	0.5980	0.6095
Gado	38:15	0.5997	0.5717	0.0007	✓	0.5976	0.5979	0.5988	0.6020	0.6022
PD	38:15	0.5968	0.5753	0.0007	✓	0.5907	0.5945	0.5965	0.5985	0.6038
FLAIR	38:15	0.5891	0.5757	0.0007	✓	0.5829	0.5872	0.5880	0.5911	0.5965
T1	43:10	0.5747	0.5747	0.7586	✓	0.5742	0.5742	0.5745	0.5750	0.5754
T2	43:10	0.5943	0.5744	0.0027	✓	0.5911	0.5930	0.5951	0.5955	0.5970
Gado	43:10	0.5664	0.5717	0.0007	✓	0.5634	0.5651	0.5672	0.5677	0.5684
PD	43:10	0.5940	0.5753	0.0007	✓	0.5888	0.5921	0.5954	0.5965	0.5973
FLAIR	43:10	0.5784	0.5757	0.9530	✓	0.5721	0.5734	0.5746	0.5830	0.5891

Table B in S2 Text. *Segmentation results are shown after excluding a defective data point (patient 26 from the original test set). The training and testing sets remain inverted following on from the previous supplementary tests. Each setup was run five times, and the Dice scores and other metrics represent the average across runs. Controls were also rerun but in the original configuration (not inverted) but without the defective datapoint.*

Supplementary data 2 (part 1)						
Modality	Dice	Ctrl	p-value	Stat-sig	Inv TPR	Ctrl TPR
T1	0.6013	0.5885	0.0002	✓	0.2529	0.2328
T2	0.5958	0.5887	0.0003	✓	0.2118	0.2472
Gado	0.6025	0.5881	0.0002	✓	0.2448	0.2326
PD	0.5964	0.5888	0.0002	✓	0.2179	0.2395
FLAIR	0.5895	0.5817	0.0036	✓	0.2092	0.2209

Supplementary data 2 (part 2)					
Inv FPR	Ctrl FPR	Inv FNR	Ctrl FNR	Inv TNR	Ctrl TNR
0.0841	0.0725	0.7471	0.7672	0.9159	0.9248
0.0819	0.0750	0.7883	0.7528	0.9181	0.9250
0.0826	0.0757	0.7516	0.7674	0.9160	0.9243
0.0816	0.0712	0.7821	0.7605	0.9184	0.9268
0.0843	0.0732	0.7908	0.7790	0.9157	0.9268

Table C in S2 Text. *Comparing Dice when training and testing on different image modalities.*

Supplementary data 3									
	Dice	Control	p-value	$p \lesssim 0.05$	Min	q1	Median	q3	Max
	T1 testing								
T2 training	0.5877	0.5856	0.0952	×	0.5856	0.5866	0.5876	0.5888	0.5899
FLAIR training	0.5906	0.5856	0.0318	✓	0.5877	0.5886	0.5902	0.5903	0.5960
PD training	0.5860	0.5856	0.3095	×	0.5842	0.5857	0.5858	0.5867	0.5875
Gado training	0.5852	0.5856	0.8413	×	0.5827	0.5831	0.5849	0.5855	0.5900
	T2 testing								
T1 training	0.5862	0.5874	0.6905	×	0.5834	0.5846	0.5867	0.5877	0.5884
FLAIR training	0.5885	0.5874	0.5476	×	0.5873	0.5874	0.5887	0.5891	0.5899
PD training	0.5862	0.5874	0.4633	×	0.5849	0.5853	0.5863	0.5871	0.5876
Gado training	0.5847	0.5874	0.0952	×	0.5828	0.5837	0.5840	0.5861	0.5871
	FLAIR testing								
T1 training	0.5649	0.5810	0.0119	✓	0.5625	0.5644	0.5650	0.5658	0.5666
T2 training	0.5637	0.5810	0.0119	✓	0.5621	0.5630	0.5633	0.5651	0.5651
PD training	0.5638	0.5810	0.0119	✓	0.5627	0.5627	0.5629	0.5634	0.5674
Gado training	0.5622	0.5810	0.0119	✓	0.5606	0.5609	0.5625	0.5625	0.5645
	PD testing								
T1 training	0.5864	0.5873	0.8412	×	0.5826	0.5866	0.5867	0.5872	0.5889
T2 training	0.5879	0.5873	0.6742	×	0.5873	0.5876	0.5878	0.5883	0.5884
FLAIR training	0.589	0.5873	0.2222	×	0.5871	0.5872	0.5894	0.5901	0.5912
Gado training	0.5837	0.5873	0.0079	✓	0.5825	0.5832	0.5833	0.5839	0.5855
	Gado testing								
T1 training	0.5850	0.5839	0.1412	×	0.5834	0.5840	0.5841	0.5860	0.5876
T2 training	0.5859	0.5839	0.05556	×	0.5845	0.5847	0.5849	0.5869	0.5886
FLAIR training	0.5885	0.5839	0.0156	✓	0.5843	0.5871	0.5901	0.5904	0.5907
PD training	0.5872	0.5839	0.0318	✓	0.5856	0.5857	0.5874	0.5880	0.5891
