# Supplementary material for: Reliability of a convolutional neural network in segmenting multiple sclerosis lesions from MRI: Impact of data augmentation, image modality and tolerance with U-Net architecture
Source: PLOS Digit Health. 2026 Apr 1;5(4):e0001316. doi: 10.1371/journal.pdig.0001316 (PMC13042652; doi:10.1371/journal.pdig.0001316)
Supplement: Attachment — Submitted filename: PLOS Article review report.docx [file pdig.0001316.s004.docx]

**Title:** Artificial Intelligence, Augmentation, Computational Methods, Dice, Machine Learning, Magnetic Resonance Imaging (MRI), Multiple Sclerosis (MS), Lesions

Restructure the abtract as shown below

**Abtract**

Background: Multiple Sclerosis (MS) is a demyelinating autoimmune disease of the central nervous system including the brain and spinal cord. Brain lesions are one of the most common radiological magnetic resonance imaging (MRI) features used both for diagnosis and clinical monitoring MS. Manually identifying and segmenting lesions is both difficult and time consuming, thus optimising approaches to reliable automatic segmentation is highly beneficial.

Objectives: The aim of this study was to assess the impact of data augmentation and manipulation on the accuracy of automated lesion segmentation using MRI scans from MS patients. Factors examined in this study include MRI modalities in both isolation and combination, image rotation, lesion size and size of testing set relative to training set.

Methods: Each factor was optimised and combined to maximise segmentation accuracy; the Dice metric was used as the focal metric to assess the efficacy of any given permutation of the setup. The statistical significance of results was assessed using the Mann-Whitney U-test and U-Net was chosen as the algorithmic method through which to segment lesions within the MRI volumes.

Results: The best Dice score achieved using the testing and training dataset as outlined in the MICCAI 2016 challenge rubric was 0.59, approximately a 2% improvement against controls. To achieve this result whilst adhering to the training and testing distribution as defined in the dataset publication, the optimal imaging sequence was determined to be proton density. The augmentation conditions used included implementing an additional rotation of the dataset (doubling it in size) and excluding lesions < 36.43 mm3 in volume.

Conclusions: A key finding of this study was that there was no statistically significant difference between using one MRI modality for training and another for testing. This suggests that a universal tool could be developed, trained on a single MRI type, and effectively applied in hospitals regardless of the specific MRI scans available to them, saving money and the time of clinicians.

**Reviewer’s Comments**

**Background**

In line 2 insert the word including after nervous system

**Objectives**

Kind state your objectives and leave out commentary

**Methodology**

Kindly summarize clearly the materials and methods used to carry out the study (Materials and methods)

**Conclusion**

Kindly link directely the objectives, methodology and results to the conclusion. What has been stated here is not the conclusion of this study

**Introduction**

Paragraph 1 Line 5 replace viewed with diagnosed

Paragraph 1 Line 6 replace “Their heterogeneity (shape, size, number, location) in

the CNS” with “The heterogeneity (shape, size, number, location) of the CNS tissues”

Replace challenges with articles

Paragraph 2 line 4 insert “the” before analysis

Paragraph 2 line 8 replace “like” with “including”

Last paragraph line 4 insert “contrast medium” after enhancing

Was any material use?

**Methods**

Briefly explain the flowchart

**Dataset**

Kindly restructure the data collection procedure with each modality under the following

1. Image acquisition
2. Protocol use
3. procedure

**Architecture**

Provide step procedure of the architecture using figure 3

**Experimental Procedure**

Where are the link with the listed equations

Any application?

**Results**

Good presentation of the result

**Discussions**

link this statement with the results tables “The main findings of this study were (1) that initial testing, using the entire dataset, indicated that Flair was the optimal image modality and Gadolinium-enhanced images produced the lowest accuracy during the automated segmentation process of lesions”

which of the results sections are you discussing? Kindly be more specific

kindly discuss the results here not commentary

**Impact of models**

Provide more information on the potential impact on the application in medical imaging.

**MS lesion segmentation specific Issues when using AI**

Good discussions

**Conclusion**

Good conclusion

**General comments**

The study comprehensively explores the impact of data adaptations in automatic MS lesion segmentation using the convolutional neural network, U-net. Using multiple MRI modalities to train a model appeared to serve no benefit in improving segmentation results although it was apparent that a model trained on one image modality was capable of segmenting lesions in another. I think the study is a good one and fairly represent the study objectives.

However, these are some observations

1. The background to the introduction was too elaborate which include some reading materials available in literature I wonder what this adds to the study.
2. The methodology should include information on the materials used and brief explanation of the flowchart
3. The datset section should be restructured to be more systematic for easy understanding
4. The must be explanation of the applications of the various equations
5. The results were presented excellently
6. The discussions must be centred on the presented results not just commentary.
7. The conclusions were fairly done and answered the study objectives

**Recommendation**

The study can be accepted for publication if the suggested corrections are done.

**References**

Check your references. It is not good enough
