# Supplementary material for: Reliability of a convolutional neural network in segmenting multiple sclerosis lesions from MRI: Impact of data augmentation, image modality and tolerance with U-Net architecture
Source: PLOS Digit Health. 2026 Apr 1;5(4):e0001316. doi: 10.1371/journal.pdig.0001316 (PMC13042652; doi:10.1371/journal.pdig.0001316)
Supplement: Attachment — Submitted filename: Response to Reviewers.docx [file pdig.0001316.s005.docx]

Dear Editors,

Thank you for the consideration of our manuscript, in addition we would like to thank the reviewers for their work in reviewing our manuscript. We, the authors, have gone through each of the comments provided by both the editors and the reviewers and have addressed each of the points presented with either amendments or rebuttal to justify the publication of our manuscript in PLOS Digital Health. In responding to each point raised, we have used the following pre-fix notation: ‘E’ for any points raised by the editor(s), and R1 for the first reviewer and R2 for the second reviewer, with the number of their specific point following this; e.g. R2.3 refers to the 3^rd^ point raised by reviewer 2.

We look forward to hearing a response.
Kind Regards,
Adam Christopher Szekely-Kohn (on behalf of all authors).

***Editors comments:***

*E1 Disclosure statement. This is published with the article. It must therefore be completed in full sentences and contain the exact wording you wish to be published.*

*i. Please clarify all sources of funding (financial or material support) for your study. List the grants (with grant number) or organizations (with url) that supported your study, including funding received from your institution.*

*ii. State the initials, alongside each funding source, of each author to receive each grant.*

*iii. State what role the funders took in the study. If the funders had no role in your study, please state: “The funders had no role in study design, data collection and analysis, decision to publish, or preparation of the manuscript.”*

*iv. If any authors received a salary from any of your funders, please state which authors and which funders.*

The Acknowledgments have been amended to emphasise that no other author, besides Adam Christopher Szekely-Kohn received funding for this study. We also acknowledge Observatoire Français de la Sclérose en Plaques (OFSEP) who collected the scan data independently of us, as per the requirement for access to their data-set. Finally, we acknowledge Bluebear, the High Performance Computing facility at the University of Birmingham, without which we would not have been able to run the computational simulations.

*E2 An ethics statement has been included stating clearly that we were not responsible for the collection the data.*

An ethics statement has been included in the methods section, stating clearly that we were not responsible for the collection of the MRI dataset used in this study.

*E3 We ask that a manuscript source file is provided at Revision. Please upload your manuscript file as a .doc, .docx, .rtf or .tex.*

We have included source .tex files along with accompanying .bib files.

*E4 Please upload separate figure files in .tif or .eps format. Also, remove the figures from your manuscript file but keep the legends.*

Images have now been uploaded in the figure format requested.

*E5. We notice that your supplementary tables are included in the manuscript file. Please remove them and upload them with the file type 'Supporting Information'. Please ensure that each Supporting Information file has a legend listed in the manuscript after the references list*.

The supplementary material has been removed from the main text as instructed with a legends list at the end of the manuscript placed after the reference list.

*E6. Thank you for uploading your study's underlying data set. Unfortunately, the repository you have noted in your Data Availability statement does not qualify as an acceptable data repository according to PLOS's standards.*

To clarify, this study uses an existing data-base available. This data-base does not belong to the authors of this study. Conditions of use include that the MRI images are not made available through other repositories and doing so violates its terms of usage (see Ethics Statement), additionally the platform used complies with European GDPR regulations. That the dataset does not belong to the authors has been clarified in the main text. We have now made a Zenodo link to the code used and added a Data Availability statement after the acknowledgements section.

*“****Data Availability***

*The dataset can be found on the Shanoir platform and can be downloaded on the following webpage: https://shanoir.irisa.fr/shanoir-ng/challenge-request. An account to facilitate the dataset download can be requested through the website. The terms and conditions relating to the use of the dataset are outlined [27], [36], [39]. The code used and instructions for its use can found in the following repository [55].”*

*E7. Some material included in your submission may be copyrighted. According to PLOS’s copyright policy, authors who use figures or other material (e.g., graphics, clipart, maps) from another author or copyright holder must demonstrate or obtain permission to publish this material under the Creative Commons Attribution 4.0 International (CC BY 4.0) License used by PLOS journals. Please closely review the details of PLOS’s copyright requirements here: PLOS Licenses and Copyright. If you need to request permissions from a copyright holder, you may use PLOS's Copyright Content Permission form.
Please respond directly to this email or email the journal office and provide any known details concerning your material's license terms and permissions required for reuse, even if you have not yet obtained copyright permissions or are unsure of your material's copyright compatibility.
Potential Copyright Issues:
Figures 2, 4, 5, and 7: Please confirm whether you drew the images / clip-art within the figure panels by hand. If you did not draw the images, please provide (a) a link to the source of the images or icons and their license / terms of use; or (b) written permission from the copyright holder to publish the images or icons under our CC-BY 4.0 license. Alternatively, you may replace the images with open source alternatives. See these open source resources you may use to replace images / clip-art:*

Figure 2, 4, 5, and 7 have been generated by uploading the MRI image data-set available via the Shanoir repository, and generating them using software (3D slicer (2,4, 5) or Python modules (7) ). The authors have written to the team responsible for the data-set, who have confirmed in writing that the generation of Figures from their MRI data-set can be disseminated in this way as per their data access agreement (which we have also uploaded). Specifically, conformation was from Romain Casey of Centre de coordination de l’OFSEP, Hôpital neurologique Pierre Wertheimer, Service de neurologie A, 59, boulevard Pinel, 69677 Bron cedex, Tél. : 04 72 12 96 41 to the first author that individual images that are from dataset can be used, as shown via the below screenshots of the written email correspondence:

However, the figure captions for Figures 2, 4, 5, and 7 have been amended to clarify that, “permission to use these images was provided by *Centre de coordination de l’OFSEP, Hôpital neurologique Pierre Wertheimer, Service de neurologie A*, in accordance with the data access agreement for the data-set used, see: [##]”.

Figure 3 has been replaced with an original see ‘U-Net Diagram’.

**Review 1 comments**

*R.1.1 Restructure the abstract as shown below:
Abstract*

*Background: Multiple Sclerosis (MS) is a demyelinating autoimmune disease of the central nervous system including the brain and spinal cord. Brain lesions are one of the most common radiological magnetic resonance imaging (MRI) features used both for diagnosis and clinical monitoring MS. Manually identifying and segmenting lesions is both difficult and time consuming, thus optimising approaches to reliable automatic segmentation is highly beneficial.*

*Objectives: The aim of this study was to assess the impact of data augmentation and manipulation on the accuracy of automated lesion segmentation using MRI scans from MS patients. Factors examined in this study include MRI modalities in both isolation and combination, image rotation, lesion size and size of testing set relative to training set.*

*Methods: Each factor was optimised and combined to maximise segmentation accuracy; the Dice metric was used as the focal metric to assess the efficacy of any given permutation of the setup. The statistical significance of results was assessed using the Mann-Whitney U-test and U-Net was chosen as the algorithmic method through which to segment lesions within the MRI volumes.*

*Results: The best Dice score achieved using the testing and training dataset as outlined in the MICCAI 2016 challenge rubric was 0.59, approximately a 2% improvement against controls. To achieve this result whilst adhering to the training and testing distribution as defined in the dataset publication, the optimal imaging sequence was determined to be proton density. The augmentation conditions used included implementing an additional rotation of the dataset (doubling it in size) and excluding lesions < 36.43 mm3 in volume.*

*Conclusions: A key finding of this study was that there was no statistically significant difference between using one MRI modality for training and another for testing. This suggests that a universal tool could be developed, trained on a single MRI type, and effectively applied in hospitals regardless of the specific MRI scans available to them, saving money and the time of clinicians.*

The abstract has been restructured and the background commentary has been reduced, the conclusion has been re-focussed and re-ordered with the methods now being streamlined and more logical. The data has now been introduced in the methods section of the abstract as opposed to results. New abstract is written below point R.1.2.

*R.1.2 Background*

*In line 2 insert the word including after nervous system*

*Objectives*

*Kind state your objectives and leave out commentary*

*Methodology*

*Kindly summarize clearly the materials and methods used to carry out the study (Materials and methods)*

*Conclusion*

*Kindly link directly the objectives, methodology and results to the conclusion. What has been stated here is not the conclusion of this study.*

*Background*

*In line 2 insert the word including after nervous system*

*Objectives*

*Kind state your objectives and leave out commentary*

*Methodology*

*Kindly summarize clearly the materials and methods used to carry out the study (Materials and methods)*

*Conclusion*

*Kindly link directely the objectives, methodology and results to the conclusion. What has been stated here is not the conclusion of this study:*

The new abstract is detailed below addressing each of the points above:

*“\textbf{\textit{Background:}} Multiple Sclerosis (MS) is an inflammatory demyelinating disease of the central nervous system, typically exhibiting radiologically identifiable lesions within the brain and spinal cord, features key to both diagnosis and clinical disease monitoring. Manually identifying and segmenting lesions is both difficult and time consuming, thus optimising approaches to reliably automate segmentation is highly beneficial. \newline*

*\textbf{\textit{Objectives:}} The aim of this study was to assess the impact of data augmentation and manipulation on the accuracy of automated lesion segmentation using MRI scans from MS patients. Factors examined in this study include MRI modalities in both isolation and combination, image rotation, lesion size and size of testing set relative to training set.\newline*

*\textbf{\textit{Methods:}} The MICCAI 2016 MS dataset was used in this study. U-Net was chosen as the algorithmic method for segmentation. Each factor was optimised and then combined to maximise segmentation accuracy; the Dice metric was used as the focal metric to assess the efficacy of any given permutation of the setup. Statistical significance was assessed using the Mann–Whitney U-test, with each permutation repeated five to ten times to ensure robustness.\newline*

*\textbf{\textit{Results:}} The best Dice score achieved using the testing and training dataset as outlined in the MICCAI 2016 challenge rubric was 0.59, approximately a 2\% improvement against controls. To achieve this result whilst adhering to the training and testing distribution as defined in the dataset publication, the optimal imaging sequence was determined to be proton density. The augmentation conditions used included implementing an additional rotation of the dataset (doubling it in size) and excluding lesions $<$ 36.43 mm$^3$ in volume. \newline*

*\textbf{\textit{Conclusions:}} The impact of data manipulation and augmentation was found to be statistically significant against controls using a Mann-Whitney U-test for lesion segmentation. An ancillary finding of this study was that there was no statistically significant difference between using one MRI modality for training and another for testing.”*

*R.1.3*

*Introduction*

*1. Paragraph 1 Line 5 replace viewed with diagnosed*

The clinical co-authors for this paper have advised that as one diagnoses a condition not an anatomical observable, this term has not been used. Instead, we have amended the wording on this line from ‘viewed’ to ‘identified’ (Multiple sclerosis is diagnosed based on the identification of a lesion).

*Paragraph 1 Line 6 replace “Their heterogeneity (shape, size, number, location) in*

*the CNS” with “The heterogeneity (shape, size, number, location) of the CNS tissues”*

The line has been amended so that it is clear that the heterogeneity of the lesions themselves are being evaluated, not the otherwise ‘healthy’ CNS tissue.

*Replace challenges with articles*

*Paragraph 2 line 4 insert “the” before analysis*

*Paragraph 2 line 8 replace “like” with “including”*

*Last paragraph line 4 insert “contrast medium” after enhancing*

The above corrections have been implemented within the manuscript.

*Was any material use?*

No further materials were used.

*1. The background to the introduction was too elaborate which include some reading materials available in literature I wonder what this adds to the study.*

Introduction:

2.

The authors agree that the introduction is elaborate, however, this is partly a consequence of tailoring the manuscript to two key audiences: firstly, those primarily interested in MS who may require further outline of machine learning, and those interested in the application of machine learning to medical imaging but may not have a background regarding MS. . The second, third, and fourth paragraphs have been made more succinct to help present a more compact introduction.

“Object identification in medical imaging has become a key challenge in modern radiology. In a clinical setting, critical judgements relating to MS diagnosis and prognosis are still made for the most part by medical professionals, reviewing imaging series directly. Heterogeneous and irregularly shaped abnormalities like MS lesions are challenging to identify. Technological advancements, specifically in artificial intelligence (AI) have led to methods by which segmentation of regions of interest (ROI), such as demyelinating lesions (or other key anatomical or pathological structures such as tumours), can be automated [11]. Deep learning techniques, such as convolutional neural networks (CNNs) have proved to be highly effective in discovering hierarchical feature representation [16]. For the segmentation of MS lesions, techniques such as graph-cuts [17], multi-layer perceptrons [18] and K-nearest neighbours [19] have been applied with high accuracy. U-Net and U-Net inspired methods have been applied successfully to segment a variety of medical objects of interest including organs like the liver, lungs, pancreas, bones, cancerous and pre-cancerous tissues [21], [22], [23], [24], [25], [26], [22] alongside central nervous system tissues and MS lesions [27].

There are published studies on MS lesion segmentation involving U-Net and the Medical Image Computing and Computer Assisted Interventions (MICCAI) dataset [28], [29], [30]. All of them achieved a better result than those which accompanied the challenge publication [14]; however, these retrospective entries did not strictly adhere to the stipulations outlined in the original challenge. For instance, large portions of the data provided was unused, volumes were deconstructed into their constituent slices, mixed, and training and testing datasets redefined on a random slice by slice basis. Only 15 of the 53 MRI volumes included in the challenge were used and these were divided between training and testing [28], [29], [30], using data from only three of the four different scanners from the MICCAI dataset. In all three cases, the individual 2665 slices comprising the fifteen MRI volumes, were split using a ratio of 1:4 for the testing and training, respectively. Hence, the trained algorithms from their studies were less likely to be generalisable, and possibly gain advantages regarding evaluation metrics which are unlikely to be reproduced in a true clinical setting. Critically, this point highlights the benefit of benchmarking the input parameters to a neural network such as U-Net.”

R.1.4. *Methods*

*Briefly explain the flowchart*

*2. The methodology should include information on the materials used and brief explanation of the flowchart*

A description of the flowchart giving an overview of the work has now been included. The methodology now contains information the 2016 MICCAI dataset and the relevant citations (Commowick 2021a) in which the dataset is written about in great detail. The U-net architecture is described briefly in the methods, however, for this reason greater explanation is included within the supplementary materials.

*R.1.5 Dataset*

*Kindly restructure the data collection procedure with each modality under the following*

*1. Image acquisition*

*2. Protocol use*

*3. procedure*

*3. The dataset section should be restructured to be more systematic for easy understanding*

The dataset section has been restructured as requested. We emphasise that a more detailed overview of the dataset collection process can be found in Commowick 2021.

*R.1.6 Architecture: Provide step procedure of the architecture using figure 3*

Figure 3 has now been amended for clarity. Additionally, its figure caption has been amended for clarity. Further, a detailed explanation the model used can now be found in the supplementary materials for Appendix I.

*R.1.7 Experimental Procedure*

*Where are the link with the listed equations*

*Any application? The must be explanation of the applications of the various equations*

The equations for the metrics used can be found in the methods, we have now signposted them in the results section by referring directly to the equation numbers.

*R.1.8 Discussions*

*link this statement with the results tables “The main findings of this study were (1) that initial testing, using the entire dataset, indicated that Flair was the optimal image modality and Gadolinium-enhanced images produced the lowest accuracy during the automated segmentation process of lesions”*

*The discussions must be centred on the presented results not just commentary.*

We have emphasised these results both in the abstract (R.1.2) and the discussion section to ensure that the results collected are discussed in deeper detail at the beginning of the discussion:

*“(1) that initial testing, using the entire dataset, indicated that FLAIR was the optimal image modality and Gadolinium-enhanced images produced the lowest accuracy during the automated segmentation process of lesions (Table~\ref{initalresults}) with Dice scores of 0.5753 and 0.5717, respectively;”*

Further to this point, we have included considerably more throughout the discussion section (see R.1.9)

*R.1.9 which of the results sections are you discussing? Kindly be more specific kindly discuss the results here not commentary*

When discussing results, reference has been made to data and results more clearly and the tables in which they can be found. Specific data has been highlighted.

In subsection “Impact of Model”

*“In this current study, it has been demonstrated that introducing additional augmentations improved segmentation illustrated by the Orig+90 FLAIR results in Table 8 where results Dice were found to improve from 0.5731 to 0.5809 when using the real dataset in combination with a single set of rotated images.”*

*“Another study used at least a portion of the dataset utilised and by using similar augmentations increased the Dice score by 6% [28]. In contrast, the augmentation undertaken in this study only increased segmentation quality by 0.5% less than a percent, from 0.5731 to 0.5809 (Table 8) .”*

In subsection “Considerations of AI in a Medical Imaging context”

*"The results improved substantially both when this volume was removed from the training and testing datasets. This can be seen firstly when comparing the sets of control data in supplementary tables 1 and 2 for every imaging modality and their respective inversion results. Control testing and inverted testing were re-run yielding an improvement of results which was statistically significant across all imaging modalities. For example, segmentation of T1 volumes, simulated in line with competition rubric \cite{Commowick2021msseg} improved from 0.5747 (Supplementary Table 1) to 0.5885 (Supplementary Table 2), similarly post inversion results for T1 images improved from 0.5970 (Supplementary Table 1) to 0.6013 (Supplementary Table 2). All results in both supplementary tables were an average of five runs and the improvement described in lesion segmentation for T1 volumes was consistent across all imaging modalities."*

*“A further set of tests were carried out to be sure that the algorithm could be both trained and tested on data produced in all centres. Interestingly, this produced worse results despite 43 MRI volumes being used in the training and only 10 in testing for all imaging modalities (e.g FLAIR Dice reducing from 0.5891 to 0.5784 over an average of five runs each).”*

In subsection “MS lesion Segmentation Specific Issues when using AI”

*“The exclusion of both of the two smaller quartiles of lesions (lesions < 36 mm3 in volume), demonstrated improved results which were statistically significant compared to the relevant control (Table 8). Dice results from Q3+Q4 for PD images were, on average 0.5844 compared to the relevant control of 0.5726 a statistically significant improvement.”*

*R.1.10 Impact of models*

*Provide more information on the potential impact on the application in medical imaging*

Further information is provided in new added clauses within the discussion section.

*“Furthermore, artificial intelligence could be utilised to highlight patients with the most malignant abnormalities allowing for a medical facility to prioritise those most in need of urgent care more effectively. This is possible because the algorithms used are trained on datasets informed by the clinical expertise of medical professionals with many years of experience.”*

*“In addition, generative machine learning models, like generative adversarial networks \cite{baronti2022artificial} and diffusion models \cite{dhariwal2021diffusion} have the capacity to standardise imaging sets produced across different institutions, scanners and annotators. A method through which this can be achieved is image-to-image translation whereby a model learns to map between image domains \cite{Isola2016}. This technique can be used to harmonise of the same species by, for instance, normalising resolution, adjusting contrast, or even converting one imaging modality to another. This results in improved consistency and interoperability across independently collected datasets.”*

*R.1.11 MS lesion segmentation specific Issues when using AI*

Addition of extra points on MS lesion specific topics related to machine learning have been incorporated into the discussion.

“The heterogeneity in lesion presentations, due to differences in patients due to demographics, disease stage or duration, variation in subtype \cite{Eshaghi2021}, can make lesion segmentation more challenging than more regularly presenting diseases such as tumours which also differ from MS in that they are masses of alien tissue as opposed to tissue scarring and thus easier to predict. The occurrence of different subtypes varies considerably with CIS, RRMS, SPMS disease course comprising the majority of MS and the PPMS course only making up 10\% to 15\% \cite{Thompson2018}. This means that there is significantly less supply of PPMS data and images for both training algorithmic models and medical students. Machine learning techniques offer an avenue for supplementing data through generative methods, enabling the production of entirely original data. This approach should be considered a subject for future work.”

*R.1.12 Amend references check your references. It is not good enough:*

We have checked the references and have added DOI links to ensure that these are correct.

**Review 2 comments**

*R.2.1 The authors chose U-Net for MS lesion segmentation, which is indeed a classic and widely used model. However, have the authors considered using nnU-Net, which is a self-adapting framework that has achieved state-of-the-art performance on various medical image segmentation tasks? A comparison or discussion would strengthen the methodology.*

This study focuses on the impact of preprocessing and alterations of data, as opposed to the model itself. Benchmarking for the use of others researchers, and models, is a key point of this study, and we leave it to those who wish to undertake future work to employ other models as a comparison, such as nnU-Net. This point is now made clear in the discussion section, to encourage others to benchmark their neural networks.

*“The information and analysis presented in this study, particularly regarding data preprocessing, the influence of MRI modalities, and data manipulation, are intended to serve as a benchmark for future research, providing a meaningful point of comparison for models used by other researchers. Further exploration using alternative neural networks is encouraged, including variations of U-Net such as nnU-Net \cite{Isensee2021}, as well as other segmentation architectures.”*

*R.2.2 The dataset used—based on the 2016 MICCAI MS Lesion Segmentation Challenge—comprises data from only 53 MS patients, which seems rather limited for training a deep learning model. Are there any additional datasets available that could be used for external validation to enhance the robustness and generalizability of the results?*

The motivation for choosing the MICCAI 2016 is that it is the most used MS dataset. There is a defined rubric for the published challenge and since this paper is essentially a bench marking paper to illustrate the impact of manipulations and augmentations, it means there are the most points of comparisons using the same dataset in the literature. Indeed, it acts as an excellent benchmark (as per the previous point), as the data-set can be viewed as an ‘industry standard’. While only 53 patients are included used, this does not mean 53 datapoints, with each brain volume being comprised of 512 slices, and given the metrics used to calculate evaluate whether a voxel has been assigned lesion or non-lesions status (Dice, TPR, TNR, FPR, FNR), this amounts to 512 × 512 × 144 ×53 datapoints for the model to evaluate. Further discussion around the limitation of smaller clinical scan studies has been added, in the context too of benchmarking.

*R.2.3 Have the authors employed 5-fold cross-validation or any other form of cross-validation during training to ensure the stability and reproducibility of the model’s performance?*

Each test performed was run between 5 and 10 times, we have now made this clear in the manuscript, and the abstract. We were able to perform our statistical analysis by virtue of the fact that each test was run multiple times and each result is an average response in which the convolutional neural network was randomly initiated each time such that results did differ slightly each time.

Abstract (methods summary)

*“Methods: The MICCAI 2016 MS dataset was used in this study. U-Net was chosen as the algorithmic method for segmentation. Each factor was optimised and then combined to maximise segmentation accuracy; the Dice metric was used as the focal metric to assess the efficacy of any given permutation of the setup. Statistical significance was assessed using the Mann–Whitney U-test, with each permutation repeated five to ten times to ensure robustness.”*

*R.2.4 Was test-time augmentation (TTA) used during inference to potentially improve segmentation accuracy? If not, the authors may consider incorporating it and reporting the impact.*

As this study already includes other forms of augmentation and does so in depth, we have decided not to pursue TTA as an additional variant. However, we now refer to further augmentation techniques, such as TTA, in the discussion as possible further studies which can be employed for benchmarking.

*“The information and analysis presented in this study, particularly regarding data preprocessing, the influence of MRI modalities, and data manipulation, are intended to serve as a benchmark for future research, providing a meaningful point of comparison for models used by other researchers. Further exploration using alternative neural networks including variations of U-Net such as nnU-Net \cite{Isensee2021}, and other segmentation architectures, other means of augmentation like test-time augmentation (TTA) \cite{shanmugam2021better} and using alternative datasets such as the ISBI 2015 \cite{Carass2017} and MICCAI 2021 \cite{Commowick2021msseg}. Comparing these benchmark results with those obtained using different architectures, augmentation techniques, and datasets can help isolate the impact of each component on segmentation accuracy, potentially informing improvements to computational frameworks under development.”*

*R.2.5 Finally, the authors should further elaborate on the potential clinical implications of applying their AI model. Specifically, how could this approach benefit clinical practice, such as improving diagnostic efficiency, reducing radiologist workload, or supporting longitudinal monitoring of disease progression?*

We have now elaborated on the clinical implication of applying our AI model to clinical practice, in addition to the information already contained.

*“This could lead to significant reductions in resource usage (MRI and clinician time) when monitoring lesion progression on any individual and improved diagnostic efficiency, as multiple scans do not need to be collected. Furthermore, machine learning models can be utilised to highlight patients with the most malignant abnormalities allowing for a medical facility to prioritise those most in need of urgent care more effectively. This is possible because the algorithms used are trained on datasets informed by the clinical expertise of medical professionals with many years of experience. This accumulated experience is then essentially being used to advise on treatment guidance and patient care, which can be especially beneficial to countries or areas with few resources and experienced medical personnel.”*

.
