# Supplementary material for: Reliability of a convolutional neural network in segmenting multiple sclerosis lesions from MRI: Impact of data augmentation, image modality and tolerance with U-Net architecture
Source: PLOS Digit Health. 2026 Apr 1;5(4):e0001316. doi: 10.1371/journal.pdig.0001316 (PMC13042652; doi:10.1371/journal.pdig.0001316)
Supplement: Attachment — Submitted filename: SecondRebuttalToCommentsPlosDH.docx [file pdig.0001316.s006.docx]

Dear Editor(s) and Reviewer,

Thank you once again for taking the time to consider our manuscript. We appreciate the additional comments and hope that the explanations provided are sufficient to merit publication in Plos Digital Health. Comments are written in **red** and the respective responses are in **blue** and changes to the manuscript are in **green**.

Kind Regards,
Adam Christopher Szekely and co-authors


Current Responses:

**Reviewer 1:** n/a

**Reviewer** 2**:** **“*The author has already addressed my concerns”***

*We thank the reviewers for their efforts in reviewing our manuscript, and are glad that we have adequately addressed their concerns.*

***Reviewer #3****: Thank you for your revision. However, the revision fails to adequately address several important concerns, particularly those highlighted by Reviewer #2. While the manuscript emphasizes data preprocessing and manipulation as its primary contribution, the current analysis does not convincingly demonstrate novelty or rigor, especially given the limited dataset and narrow scope of augmentation techniques. Below, I detail a few major concerns.*

Firstly, we thank the reviewer for re-reviewing our manuscript. Secondly, we politely disagree with Reviewer-3 on whether points raised by other reviewers have been addressed; in particular, we note that Reviewer-2 is also satisfied by our revisions (verbatim, “***The author has already addressed my concerns***”).

More broadly, the U-Net model, which underpins many contemporary segmentation approaches, offers a practical advantage for this study (Ronneberger 2015). Its established and well-understood architecture allows the effects of data type changes and data manipulations to be assessed directly, without confounding influences arising from model-specific design choices. Below, we have aimed to address each point raised.

*1. The author used a very basic U-Net model and did not provide a comparison with recent best-in-class models. Preprocessing effects cannot be interpreted meaningfully unless the underlying model is competitive and representative of current standards. When the author mentioned an improvement of 2% over MICCAI2016, did the author compare the model performance on the same footing, like excluding lesions < 36.43 mm³ in volume and well-stratified data in train, val, and test? Also, it would be helpful if the author can further clarify on threshold-deciding criteria for lesion size exclusion.*

The objective of this work is not to compare segmentation models, but rather to analyse the impact of data types and data augmentation strategies on segmentation performance. Introducing model architecture as an additional variable would place the study outside its intended scope, the paper is also already 23 pages long, excluding the figures (which have be removed at the request of the journal for the editing process). Model comparison is already well explored in the literature; however, comparatively few studies focus specifically on how data representation and augmentation influence downstream performance, which is the primary contribution of this work. Furthermore, as the U-Net model employed is the best understood, and mostly compared, it enables the most robust assessment of the variables evaluated. In addition, a table of published results using the same dataset using a variety of models is included as a point of comparison (see Table 9).

*“Further exploration could involve alternative neural network architectures, including variations of U-Net such as nnU-Net \cite{Isensee2021}, as well as other segmentation models. Additional data augmentation strategies, such as test-time augmentation (TTA) \cite{shanmugam2021better}, and the use of alternative datasets, including ISBI 2015 \cite{Carass2017} and MICCAI 2021 \cite{Commowick2021msseg}, also merit investigation. Systematic comparison of benchmark results across different architectures, augmentation techniques, and datasets may help to isolate the contribution of each component to segmentation accuracy, thereby informing potential improvements to computational frameworks under development. While direct generalisation cannot be assumed, the findings of this study are nonetheless expected to be broadly applicable to more recent iterations of U-Net and other convolutional neural networks with similar architectural principles such as the encoder–decoder structure, skip connections and convolutional feature hierarchies.”*

The thresholding criteria can be clarified as follows. Connected lesion components (“islands”) were identified across all volumes, and their individual volumes quantified. The total number of lesions identified was x. These lesions were then ranked by volume and partitioned into quartiles: the smallest 25% defined Q1, the next 25% Q2, and so on. This stratification enabled a systematic investigation of lesion size effects. We made a included a clarification in the new manuscript at reviewer 3’s request. The entire dataset (testing and training were all included in this process

*“The rationale for the quartile distribution was that 2047 continuous lesion islands were present across the 53 subjects in the dataset varying in size from 1.38 mm$^3$ to 66300.60 mm$^3$. This was determined using a python script used to count and quantify the lesions through the processing of the lesion masks within dataset. The lesions were first ranked in ascending order by size across all patients. The smallest 512 lesions were assigned to Q1, the next 512 lesions (i.e. the 513$^{th}$ to 1024$^{th}$ lesions) were assigned to Q2, and subsequent quartiles were defined analogously. Lesion quartile assignment was performed on the combined dataset, including both training and testing data, rather than treating them independently.”*

*2. The data is limited, though it has multiple frame in one image; the author should produce 3-fold cross-fold validation or provide external validation set-based comparison for the current model and other state-of-the-art models. Re-running the same train/test split is not cross-validation and is unable to assess generalizability.*

The dataset was collected across four separate centres and four separate hospitals using different MRI scanners. From a medical imaging perspective, this is towards the top-end of what any reasonable, well-funded, study could expect. It is a limitation which needs to be accounted for by machine learning studies, if there are to be meaningful advanced in digital healthcare technology which can be transferred from lab to benchside. Indeed, the MICCAI data set was specifically sought out as it is provided by the *Medical Image Computing and Computer Assisted Intervention* (MICCAI) Society. Briefly, five separate imaging modalities were rigorously and near exhaustively analysed, with four different sets of analyses being undertaken. The MICCAI 2016 dataset is comprised of 53 patient brain volumes, each of these volumes is comprised of 512 2D slices. In total for a single modality (FLAIR for example), there are 512 × 53 unique 2D images contained within the dataset (27,136). Considering there are five versions of this dataset due to the five imaging modalities, this amounts to 135,680 2D images. The ground truth mask for the lesion delineations used were an amalgamated product of seven independent raters (with relevant clinical training) with exclusions made for outliers during the process used to combine them. This makes this particular dataset highly heterogenous both in its imaging content and the ground truth.

*“As with any machine learning study, there is always scope to include greater variability and increase the study numbers. However, to ensure a robust study a large dataset comprising 53 patients was chosen. Each of the volumes was made up of 512 2D slices meaning 27,136 (512 $\times$ 53) unique 2D images. Given that there are five versions of the dataset due to the five imaging modalities, this amounts to 135,680 2D images. In terms of variability, the dataset was collected across four different centres using four differ MRI scanners using different protocols for each of modalities. In addition, the ground truth segmentation for the MICCAI 2016 MS dataset product of seven independent raters with relevant clinical training with exclusions made for outliers during the curation process when combining them. All of the aforementioned points make this particular dataset ideal for this study.”*

*3. Combining multiple MRI modalities in training did not yield a clear benefit over the best single modality. The majority of multi-modal combinations produced no statistically significant improvement in Dice. The authors conclude that ’dataset hybridisation’ is not beneficial and the reason could be that the model had limited capacity or training data to exploit the extra information, or that some modalities introduced noise/redundancy. However, the author does not cross-check the results with other AI models or augmentations, which leads to contrasting findings and weakens the claims.*

This study was not designed to benchmark machine learning models, but rather to examine how changes to the data itself, through modality selection, combination, and augmentation, impact segmentation performance when using a fixed baseline model. Each experimental factor, imaging modality, modality combinations, rotation-based augmentation, and lesion size restriction, was deliberately examined in isolation, with all other variables held constant. This controlled design enables the specific impact of each factor to be assessed independently, without confounding effects from changes in model architecture or optimisation strategy. The findings from this study are directly applicable to clinical practice, and medical centres with imaging facilities, as it evidences the ability to use a single modality of imaging for detection of lesions when performed using AI technologies.

*“The similar lesion segmentation performance observed across MRI modalities has the potential to enable significant reductions in resource usage and financial cost, including MRI acquisition time and clinician workload, when monitoring lesion progression in individual patients. This may also improve diagnostic efficiency by reducing the need for multiple scans, thereby highlighting the potential utility of deploying machine learning–based segmentation models in hospital settings.”*
